# Angiotensin II inhibits apoptosis of mouse aortic smooth muscle cells through regulating the circNRG-1/miR-193b-5p/NRG-1 axis

**DOI:** 10.1038/s41419-019-1590-5

**Published:** 2019-05-01

**Authors:** Yan Sun, Suli Zhang, Mingming Yue, Yang Li, Jing Bi, Huirong Liu

**Affiliations:** 1 Department of Physiology & Pathophysiology, School of Basic Medical Sciences, Capital Medical University, 10 Xitoutiao, You An Men Street, 100069 Beijing, P. R. China; 2 Beijing Key Laboratory of Metabolic Disorder Related Cardiovascular Disease, Capital Medical University, 100069 Beijing, P. R. China

**Keywords:** Apoptosis, Non-coding RNAs

## Abstract

Angiotensin II (Ang II) is known to promote proliferation of vascular smooth muscle cells (VSMCs) in vascular remodeling, but whether it has an anti-apoptotic effect needs to be explored. Neuregulin-1 (NRG-1) as a member of the epidermal growth factor family was reported to suppress the proliferation of VSMCs by activating ErbB receptors, and therefore we hypothesized that there might be a cross talk between the anti-apoptotic effect of Ang II and the anti-proliferative effect of NRG-1 in VSMCs. The aim of the present study was to observe the expression and role of NRG-1 underlying the inhibitory effect of Ang II on apoptosis of mouse aortic smooth muscle cells (MASMCs). It was found that NRG-1 expression was down-regulated via the circNRG-1/miR-193b-5p-mediated post-transcriptional mechanism in response to Ang II. In addition, NRG-1 overexpression reversed the inhibitory effect of Ang II on apoptosis in MASMCs. Our data may provide a molecular basis for further understanding the mechanism of Ang II in suppressing the apoptosis of MASMCs by decreasing NRG-1 expression at circular RNA and micro RNA levels. The circNRG-1/miR-193b-5p/NRG-1 axis may prove to be a potential target for Ang II to inhibit the apoptosis of VSMCs and lead to vascular remodeling.

## Introduction

Vascular remodeling is a pathophysiological process in many cardiovascular diseases, such as atherosclerosis and hypertension^1^. Increasing evidence has demonstrated that proliferation and apoptosis of vascular smooth muscle cells (VSMCs) are key events in vascular remodeling^2^. Changes of the renin-angiotensin-aldosterone system (RAAS) may alter the balance between proliferation and apoptosis of VSMCs^3,4^. Angiotensin II (Ang II) is an effector peptide of the RAAS, and also a modulator of VSMC growth with proliferation/apoptosis effects mediated by activation of Ang II type 1 receptor (AT_1_R) or Ang II type 2 receptor (AT_2_R)^5^. In the early stages of vascular remodeling, Ang II promoted proliferation^6,7^ but inhibited apoptosis^8,9^ of VSMCs. A fundamental strategy for the treatment of these cardiovascular diseases is to accelerate apoptosis of VSMCs^10^. It is therefore important to gain insights into details of the molecular mechanism of Ang II in inhibiting apoptosis of VSMCs.

Neuregulin-1 (NRG-1) is a member of the epidermal growth factor (EGF) family, whose isoforms can be produced from the NRG-1 gene by alternative splicing^11^. Its transmembrane isoform includes an extracellular domain with an EGF-like sequence (NRG-1-ECD) and a highly conserved intracellular domain (NRG-1-ICD)^12^. NRG-1-ECD is a bioactive fragment, which can bind to ErbB family receptor tyrosine kinases to activate ErbB signaling in target cells^13^. NRG-1-ICD can translocate into the nucleus to regulate the gene expression, which has been confirmed by our previous and other studies^14,15^. Several lines of evidence have demonstrated that NRG-1 plays important roles in vascular physiopathology^16^. First, NRG-1 is expressed in vascular endothelial cells and smooth muscle cells, and its receptors are localized to the underlying smooth muscle cells^17^. Second, treatment of cultured VSMCs with NRG-1-ECD significantly decreases platelet-derived growth factor (PDGF)-BB-stimulated proliferation and migration^18^. Third, NRG-1-ICD is stimulated by transforming growth factor-β1 (TGF-β1), and translocates into the nucleus to regulate the α-SMA gene expression in human aortic smooth muscle cells (HASMCs)^15^. Previous studies showed that Ang II decreased NRG-1-ECD expression in endothelial cells^19^. However, the role of NRG-1 in the regulation of VSMC apoptosis in the context of Ang II signaling remains unclear.

Circular RNAs (circRNAs), which are shown to be a kind of critical gene regulator, are a novel class of non-coding RNAs with the characteristic of covalent bond linking the 3′ and 5′ ends generated by back splicing^20^. CircRNAs are known to play roles in the regulatory networks governing gene expression with multi-functions, such as cytoplasmic miRNA sponges^21^, RNA-binding protein participants^22^, and nuclear transcriptional regulators^23^. For example, one abundant circHIPK3, which regulates cell growth in cancerous tissues, acts as miRNA sponges to inhibit miR-124 activity by directly binding to miR-124^24^. The exon-intron circular RNAs circEIF3J and circPAIP2 interact with U1 snRNP and enhance transcription of their parental genes in the nucleus via specific RNA-RNA interaction^25^. However, the function of circRNAs under the treatment of Ang II in VSMC apoptosis remains unknown.

In this study, we showed that Ang II inhibited apoptosis through decreasing the expression of NRG-1 in mouse aortic smooth muscle cells (MASMCs), and this inhibitory effect could be reversed by NRG-1. In addition, circNRG-1 targeted NRG-1 for degradation by binding to miR-193b-5p. The results of the present study revealed a novel anti-apoptosis mechanism of Ang II via the circNRG-1/miR-193b-5p/NRG-1 axis, which may provide a potential therapeutic strategy for the prevention and treatment of vascular remodeling diseases.

## Materials and methods

### Cell culture and treatment

MASMCs (ATCC, No.CRL-2797TM) were cultured in low-glucose Dulbecco’s modified Eagle’s medium (DMEM) containing penicillin, streptomycin and 10% fetal bovine serum (FBS). Human embryonic kidney 293A cells were obtained from ATCC (Manassas, VA) and maintained in high-glucose DMEM supplemented with 10% FBS. The cells were all in a humidified incubator at 37 °C with 5% CO_2_. Before stimuli and infection with adenovirus vectors or plasmids, MASMCs were incubated in serum-free medium for 24 h. Angiotensin II (A9525), Losartan (6188) and PD123319 (P186) were purchased from Sigma-Aldrich.

### Cell apoptosis assay

MASMC apoptosis assays were performed with the Annexin V-FITC Apoptosis Detection Kit (KeyGEN BioTECH) according to the manufacturer’s recommendations. Cells were trypsinized, washed with cold PBS twice, re-suspended in 500 μl binding buffer containing 5 μl annexin V-FITC and 5 μl PI, and cultured at room temperature in the dark for 15 min. Finally, apoptosis was detected with a flow cytometer. All groups were evaluated in a minimum of three separate wells per experiment.

### Western blot analysis

Proteins from cultured MASMCs were prepared as previously described^15^. Proteins were separated on 8%, 10% or 12% SDS-PAGE gels, and transferred to PVDF membranes. The membranes were blocked with 5% milk in TTBS for 2 h at room temperature and incubated with primary antibodies overnight at 4 °C. Antibodies are as follows: anti-NRG-1 (1:500, sc-348, santa), anti-NRG-1-ICD (1:500, sc-393009, santa), anti-Cleaved-caspase-3 (1:5000, ab214430, abcam), anti-caspase-3 (1:2000, ab184787, abcam), bax (1:1000, ab32503, abcam), bcl-2 (1:2000, ab182858, abcam), p-ErbB_2_ (1:500, ab47262, abcam), ErbB_2_ (1:500, ab131490, abcam), p-ErbB_3_ (1:1000, ab133459, abcam), ErbB_3_ (1:100, ab5470, abcam), p-ErbB_4_ (1:1000, ab76132, abcam), ErbB_4_ (1:1000, ab76303, abcam) and anti-β-actin (1:1000, sc-47778, Santa Cruz). The membranes were washed, incubated with the HRP-conjugated secondary antibodies for 1 h at room temperature, treated with the Immobilon™ Western (Millipore) and detected by ECL (enhanced chemiluminescence) BIO-RAD (721BR10829). All experiments were replicated three times.

### Isolation of RNA and PCR

MASMCs were lysed by using the QIAzol Lysis Reagent (QIAGEN, Catalog no.79306). RNA was extracted from the above sample using a miRNeasy Mini Kit (QIAGEN, Catalog no.217004) according to the manufacturer’s instructions. The quality of the RNA was determined using a NanoPhotometer P-Class (IMPLEN). For large mRNA and circRNA, cDNA was synthesized using a M-MLV First Strand Kit (Life Technologies) with random hexamer primers. Quantitative real-time PCR (qRT-PCR) of mRNAs or circRNAs was performed using Platinum SYBR Green qPCR Super Mix UDG Kit (Invitrogen). For microRNA, reverse transcription and qRT-PCR were performed using the miRNA Detection Kit (Genepharma, Shanghai, China) and internal control U6 according to the manufacturer’s protocol. qRT-PCR was carried on an ABI 7500 FAST system (Life Technologies). Relative amounts of transcripts were normalized with GAPDH and calculated using the 2^−ΔΔCt^ formula as previously described^26^. For reverse transcriptional PCR (RT-PCR) of mRNA and circRNA, 5 μl 1:5 diluted cDNA or gDNA was amplified (22–37cycles, depending on the template) in a 25 μl PCR reaction using the KOD XtremeTM HotStart Polymerase Kit (71975-3, Novagen). The experiments were carried on a Thermal Cycler Block 5020 (Thermo Fisher). The primer sequences were as follows: mouse GAPDH, 5′-AAGGTGAAGGTCGGAGTC-3′ and 5′- GATTTTGGAGGGATCTCG-3′; mouse NRG-1, 5′- GGAGATGCGAGCATAGACCG-3′ and 5′-GTGTCTCGGGGCTACTCTTG-3′; mouse circNRG-1, 5′-AACCCCTGACTCCTACAGAGACT-3′ and 5′- CTGGTCCCAGTCGTGGATGT-3′.

### Microarray analysis

Circular RNA expression profiling was performed using Arraystar Mouse circRNA Array V2 analysis (Arraystar, USA). Circular RNAs of MASMCs were extracted using QIAzol Lysis Reagent (QIAGEN, Catalog no.79306) according to the manufacturer’s instructions. The sample preparation and microarray hybridization were performed based on the Arraystar’s standard protocols. Briefly, total RNAs were digested with Rnase R (Epicentre, Inc.) to remove linear RNAs and enrich circular RNAs. Then, the enriched circular RNAs were amplified and transcribed into fluorescent cRNA utilizing a random priming method (Arraystar Super RNA Labeling Kit; Arraystar). The labeled cRNAs were hybridized onto the Arraystar Mouse circRNA Array V2 (8 × 15K, Arraystar). After washing slides, the arrays were scanned by the Agilent Scanner G2505C. Agilent Feature Extraction software (version 11.0.1.1) was used to analyze acquired array images.

### Fluorescence in situ hybridization

For circRNA FISH, cells were fixed in 4% paraformaldehyde for 5 min at room temperature, then permeabilized with 0.5% Triton X-100 and washed with PBS. The process used a Ribo^TM^ Fluorescent In Situ Hybridization Kit (RiboBio, China). In brief, MASMCs were blocked with pre-hybridization buffer for 30 min at 37 °C and then incubated with a circNRG-1 FISH Probe Mix and hybridization buffer overnight at 37 °C. Cell slides were washed with hybridization washing buffer I, II, and III for 15 min at 42 °C and 0.2 × SSC at 53 °C.

The double FISH assay was performed as described previously^15^. The procedures were conducted with a miR-193b-5p double-fluorescein (both 5′- and 3′- were labeled with FITC) FISH probe (Genepharma, China) and a cy3-circNRG-1 FISH probe. Hybridization was performed using fluorescence-labeled probes in hybridization buffer by incubation at 55 °C for 1.5 h. After stringent washing with SSC buffer, then DAPI (157574, MB biomedical) was used for nuclear counter staining. Images were captured by confocal microscopy and processed by LAS AF software.

### Luciferase assay

Human embryonic kidney 293A cells were maintained as previously described^27^. For luciferase assays, HEK 293A cells were transfected with miR-193b-5p mimic (Gene pharma; Shanghai) and NC mimic combined with luciferase reporter plasmid of circNRG-1, circNRG-1 mut, NRG-1 3′-UTR, NRG-1 3′-UTR mut or an empty vector. Cells were harvested and luciferase activity was measured using a dual luciferase assay kit (Promega) after 24 h. The specific target activity was expressed as the relative activity ratio of firefly luciferase to renilla luciferase. All constructs were evaluated in a minimum of three separate wells per experiment.

### Transfection of siRNA, plasmid, miRNA, and adenovirus vectors

Small interfering RNAs (siRNAs) targeting mouse circNRG-1 (si-circNRG-1) was designed and synthesized by RiboBio (Guangzhou, China). The siRNA sequence was as follows: circNRG-1 siRNA (si-circNRG-1), 5′-ATAGTGAAAGCCACATCTA-3′. Non-specific siRNA (si-Control) was purchased from RiboBio. The plasmid of circNRG-1 (pLVX-circNRG-1) was entrusted to Likely Biotechnology (Beijing, China). Sequences of circNRG-1 and NRG-1 gene 3′-UTR contain miR-193b-5p target sites or its mutant sequences were inserted into pmir-GLO Dual-Luciferase miRNA Target Expression Vector. miR-193b-5p mimic, inhibitor and control RNAs were designed and synthesized by Gene Pharma (Shanghai, China). Adenoviruses encoding NRG-1 (Ad-GFP-NRG-1) and GFP control (Ad-GFP) were entrusted to Hanbio (Shanghai, China). Transfection was performed using Lipofectamine 2000 following the manufacturer’s instructions. Twenty-four hours after transfection, MASMCs were treated with Ang II, miR-193b-5p-mimic, or anti-miR-193b-5p, harvested and lysed for Western blot, PCR and luciferase assay.

### Statistical analysis

All the data are presented as the means ± S.E.M. Differences between two groups were assessed using analysis of variance followed by a Student’s *t*-test. A value of *P* < 0.05 was considered statistically significant. Statistical analysis was performed using Graphpad Prism 5 software (GraphPad Software, San Diego, CA, USA).

## Results

### Ang II decreases NRG-1 protein expression in MASMCs

**Fig. 1:**
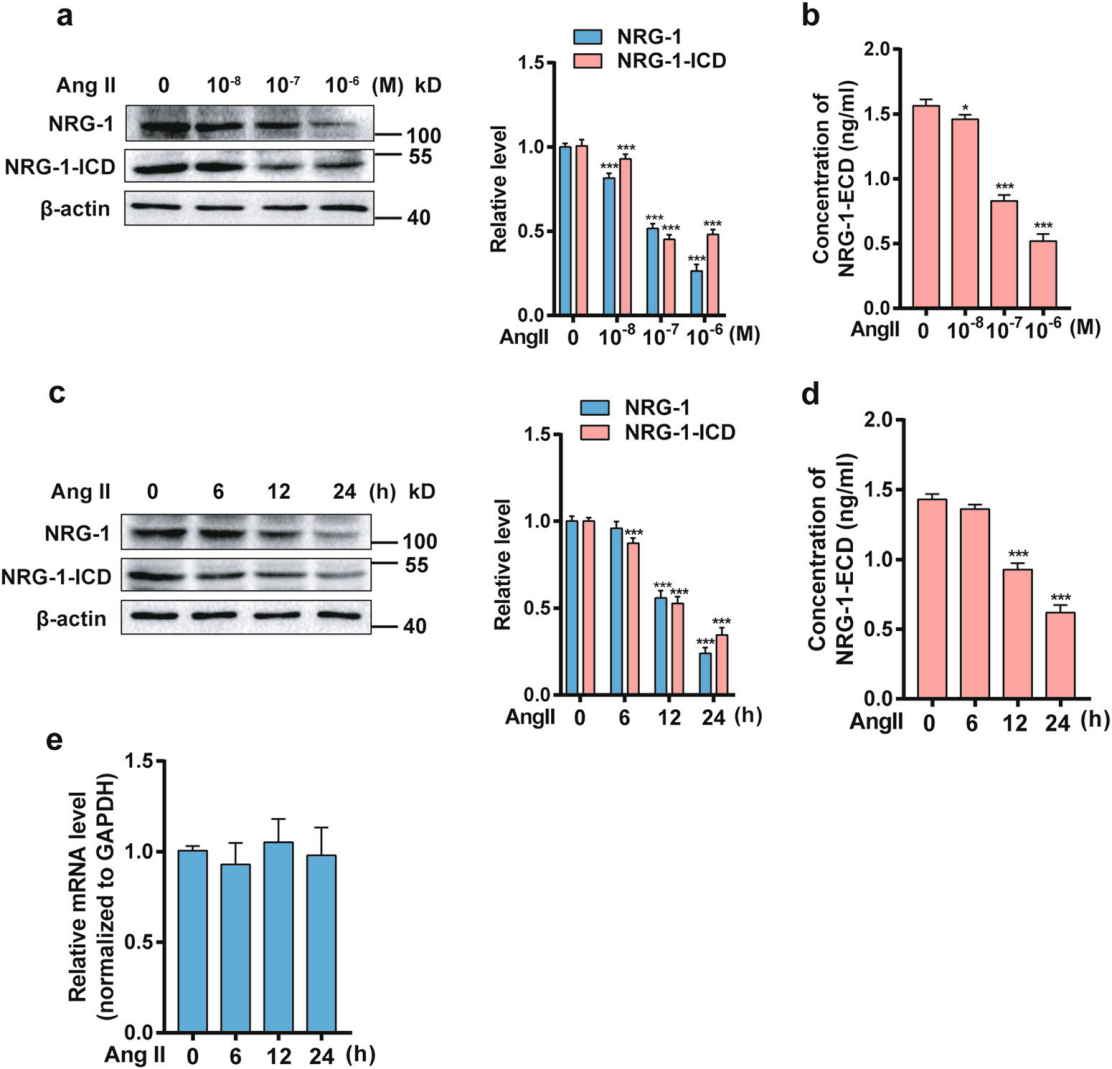
NRG-1 is down-regulated in response to Ang II in MASMCs. **a** NRG-1 and NRG-1-ICD expression levels in MASMCs treated with Ang II with different doses were analyzed by Western blot (left). The right panel shows densitometric analyses from three independent experiments. ***P < 0.001 *vs*. Ang II for 0 M. **b** Levels of NRG-1-ECD expression in the culture medium of MASMCs treated with different doses of Ang II were determined by ELISA. *P < 0.05, ***P < 0.001 *vs*. Ang II for 0 M. **c** Western blot was used to assess the NRG-1 and NRG-1-ICD expression levels in MASMCs treated with Ang II (10^−7^ M) for the indicated times (left). The right panel shows densitometric analyses from three independent experiments. ***P < 0.001 *vs*. Ang II for 0 h. **d** Levels of NRG-1-ECD expression in medium treated with Ang II (10^−7^ M) for the indicated times were analyzed by ELISA. ***P < 0.001 *vs*. Ang II for 0 h. **e** MASMCs were treated with Ang II (10^−7^ M) for the indicated times, and NRG-1 mRNA was detected by qRT-PCR. Data represent the means ± SEM of three independent experiments

To examine the effect of Ang II on NRG-1, we firstly analyzed the expression of NRG-1 in MASMCs treated with Ang II. Knowing that NRG-1 included an extracellular domain (ECD) and an intracellular domain (ICD), we first detected the expression levels of full-length NRG-1, NRG-1-ICD, and NRG-1-ECD, respectively. Western blot showed that Ang II significantly decreased both full-length NRG-1 and NRG-1-ICD protein levels in a dose- and time-dependent manner (Fig. 1a, c). Furthermore, there was a reduction in NRG-1 from cell membrane lysates and accumulation of NRG-1-ECD in the media from MASMCs in response to Ang II (Fig. 1b, d). Next, we sought to determine whether Ang II decreased the transcription of NRG-1 gene. MASMCs were treated with Ang II 10^−7^ M for different times and then NRG-1 mRNA was detected by qRT-PCR. Interestingly, we found that NRG-1 mRNA level was not affected by Ang II treatment (Fig. 1e). Based on these findings, we hypothesized that Ang II reduced protein expression of NRG-1 through post-transcriptional regulation.

### Ang II suppresses circNRG-1 expression in MASMCs

**Fig. 2:**
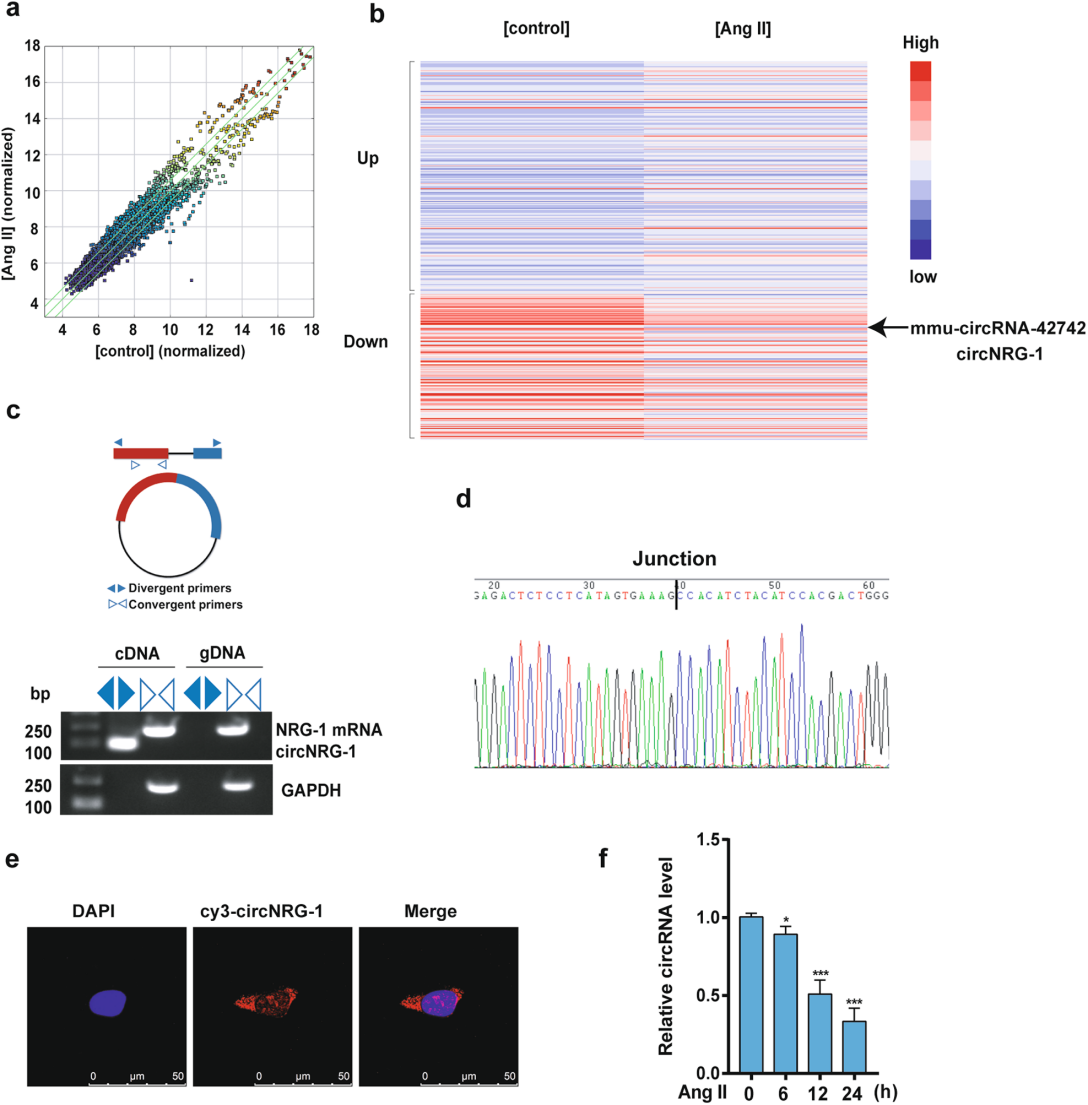
circNRG-1 is expressed in MASMCs and decreased in response to Ang II. **a** Scatter plots were used to evaluate the difference in the expression of circRNAs between Ang II and control groups. The values plotted on X and Y axes are the averaged normalized signal values of each group (log2 scaled). The circRNAs above the top green line and below the bottom green line indicate >1.5-fold change between the two groups. **b** Hierarchical clustering analysis showed the differentially expressed circRNAs over 2.0-fold change. Red color indicates high expression level, and blue color indicates low expression level. **c** Divergent and convergent primers were used to verify whether circNRG-1 was a circRNA. Convergent primers were used to detect NRG-1 mRNA. Divergent primers amplified circNRG-1 in cDNA but not gDNA. GAPDH served as linear control and size marker in base pairs. **d** Sanger sequencing confirmed head-to-tail junction of circNRG-1. **e** RNA fluorescence in situ hybridization for circNRG-1 was detected. Nuclei were stained with DAPI. Scale bars = 50 μm. **f** qRT-PCR detected circNRG-1 expression in MASMCs treated with Ang II (10^−7^ M) for the different times. Data represent the means ± SEM of three independent experiments. *P < 0.05, ***P < 0.001 *vs*. Ang II for 0 h

The finding that Ang II downregulated NRG-1 protein level without affecting mRNA level suggested that Ang II regulated NRG-1 expression post-transcriptionally via miRNAs or circRNAs. An Arraystar mouse circRNA Microarray was accomplished with MASMCs to detect whether circRNAs were involved in the regulation of NRG-1. The differential expression levels of circRNAs between Ang II and control groups were identified through scatter plots (Fig. 2a). In the scatter plots, circRNAs above the top green line and below the bottom green line showed >1.5-fold change between the two compared groups. A heat map of 382 circRNAs was differentially expressed between Ang II and control groups. Among the 382 circRNAs, 235 were upregulated and 147 were downregulated by >2-fold change in Ang II group. The results showed that mmu-circRNA-42742, whose parental gene is NRG-1, was significantly down-regulated in the MASMCs treated with Ang II as compared with the control group (Fig. 2b). To further validate the results, we used convergent and divergent primers, respectively, to amplify total RNA and circular RNA transcripts derived from the NRG-1 gene by RT-PCR (Fig. 2c). Its PCR products were confirmed by DNA sequencing (Fig. 2d). The result of fluorescence in situ hybridization (FISH) showed that circNRG-1 mostly located in the cytoplasm of MASMCs (Fig. 2e). A general consistency was shown between the results of qRT-PCR and microarray analysis. The results showed that circNRG-1 was decreased in a time-dependent manner in the group treated with Ang II in MASMCs (Fig. 2f). These data suggested that Ang II reduced circNRG-1 expression.

### circNRG-1 upregulates NRG-1 expression by acting as miR-193b-5p sponge

**Fig. 3:**
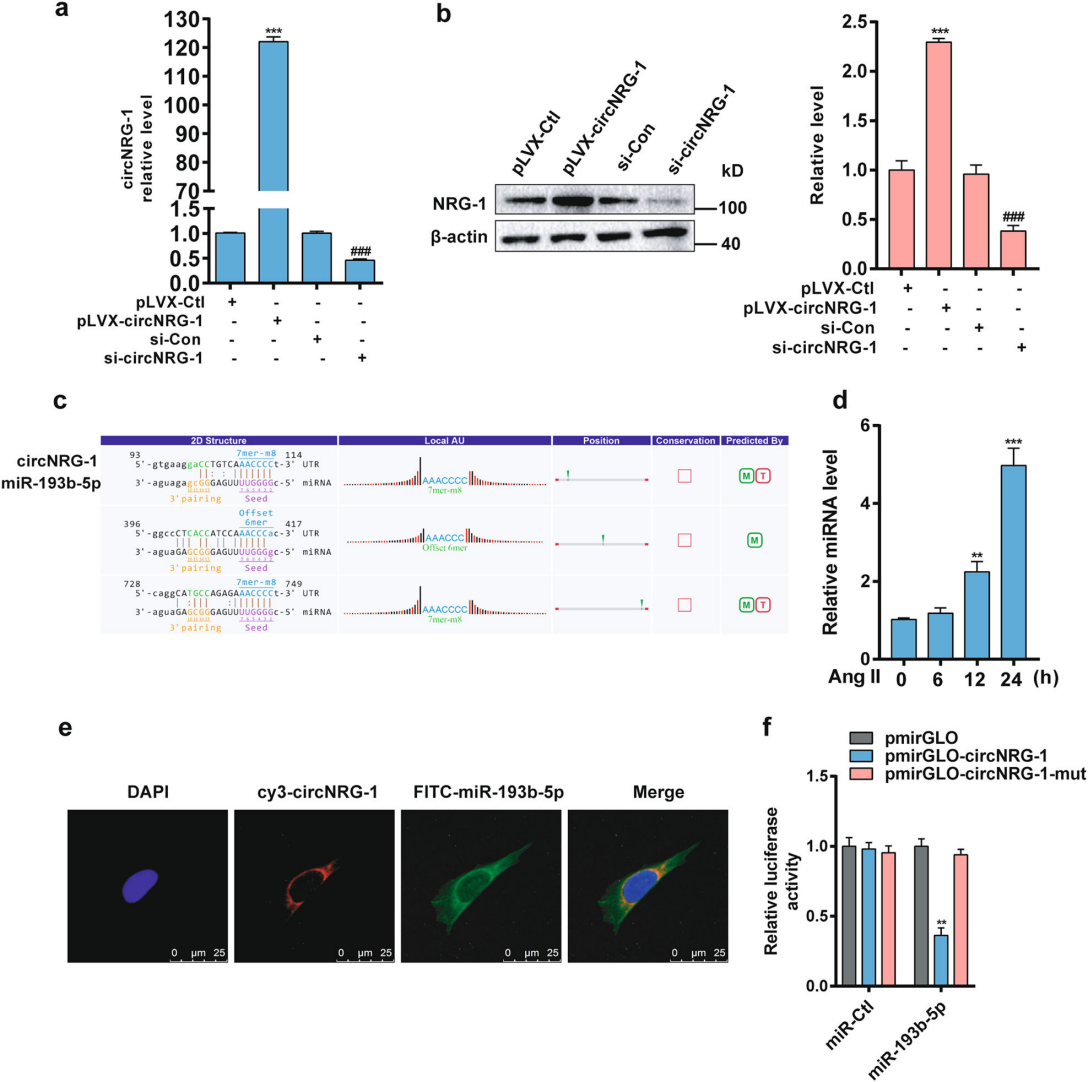
circNRG-1 acts as a miR-193b-5p sponge to regulate NRG-1 expression. **a** MASMCs were transfected with pLVX-circNRG-1 or si-circNRG-1, as well as with their corresponding controls. circNRG-1 expression was analyzed by qRT-PCR. ***P < 0.001 *vs*. pLVX-Ctl, ###*P* < 0.001 *vs*. si-Con. **b** MASMCs were transfected with pLVX-Ctl, pLVX-circNRG-1, si-Con and si-circNRG-1 for 24 h. NRG-1 was quantitated by densitometry and values were normalized to total β-actin (Right panel). Data were means ± SEM, *n* = 3. ***P < 0.001 *vs*. pLVX-Ctl, ###*P* < 0.001 *vs*. si-Con. **c** miR-193b-5p binding sites in the circNRG-1 sequence were predicted. **d** miR-193b-5p level in MASMCs treated with Ang II (10^−7^ M) for the indicated times was examined by qRT-PCR. Data are presented as means ± SEM, *n* = 3. **P < 0.01, ***P < 0.001 *vs*. 0 h. **e** Co-localization between circNRG-1 and miR-193b-5p was detected by RNA in situ hybridization in MASMCs. Nuclei were stained with DAPI. Scale bars = 25 μm. **f** HEK 293A cells were co-transfected with wild-type pmirGLO-circNRG-1 or its mutant pmirGLO-circNRG-1 mut and miR-193b-5p mimic or miR-Ctl. Luciferase activity was measured. Data are the means ± SEM of three independent experiments. **P < 0.01 *vs*. pmirGLO, pmirGLO-circNRG-1 mut

Knowing that Ang II decreased the expression levels of NRG-1 and circNRG-1, we sought to determine the relationship between them by overexpressing or knocking down circNRG-1 in MASMCs. Knowing that the exogenous circNRG-1 was effectively overexpressed in pLVX-circNRG-1-transfected MASMCs, we designed a siRNA targeting the backsplice sequence for circNRG-1 (si-circNRG-1) as shown by qRT-PCR (Fig. 3a). Western blot analyses revealed that circNRG-1 overexpression or knockdown markedly increased or decreased NRG-1 protein level, respectively (Fig. 3b). Knowing that circRNAs can function as miRNA sponges, we predicted miRNAs, whose nucleotide sequences are known to be complementary to a region of circNRG-1, and found out their interactions. The circRNA/miRNA interaction was predicted with Arraystar’s home-made miRNA target prediction software based on TargetScan and miRanda. The result showed that circNRG-1 contained sequences complementary to miR-193b-5p seed sequence (Fig. 3c). In addition, Ang II significantly increased the miR-193b-5p level (Fig. 3d). Localization of miR-193b-5p and circNRG-1 was showed by RNA in situ hybridization in MASMCs, and their co-localization was predominantly in cytoplasm (Fig. 3e). To further clarify whether the sponge effect of circNRG-1 on miR-193b-5p affected gene expression, circNRG-1 sequence was inserted immediately in downstream of the luciferase reporter gene. Luciferase assay revealed that transfection with miR-193b-5p mimic significantly decreased the luciferase activity via mediation of wild-type circNRG-1 sequence but not via its mutant (Fig. 3f). These findings suggested that circNRG-1 regulated NRG-1 expression and bound miR-193b-5p to prevent it from binding to the target genes.

### miR-193b-5p suppresses NRG-1 expression by targeting its 3′-UTR in MASMCs

**Fig. 4:**
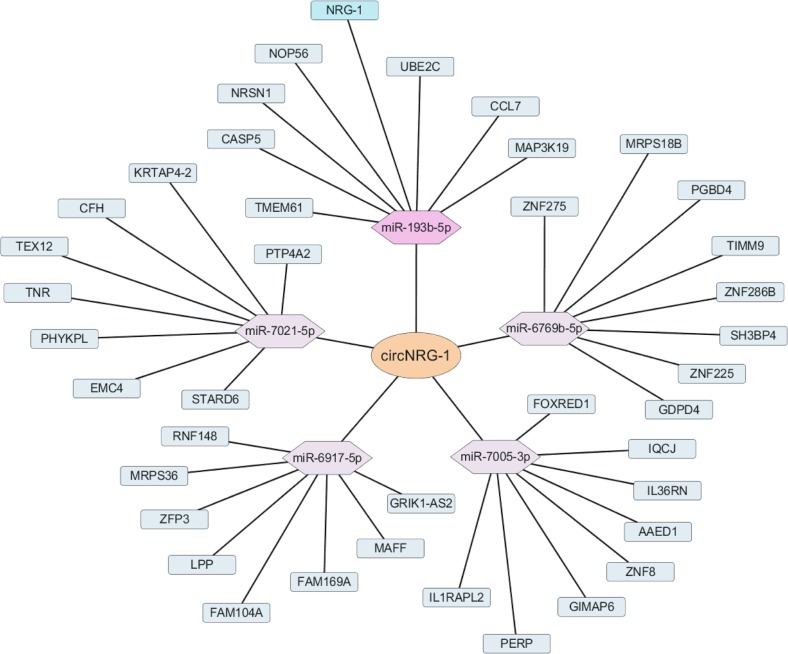
circRNA-miRNA-mRNA regulatory network. The orange, purple and green nodes represent circRNA, miRNA and mRNA respectively. Markers highlighting staining showed circNRG-1-miR-193b-5p-NRG-1 interactions

**Fig. 5:**
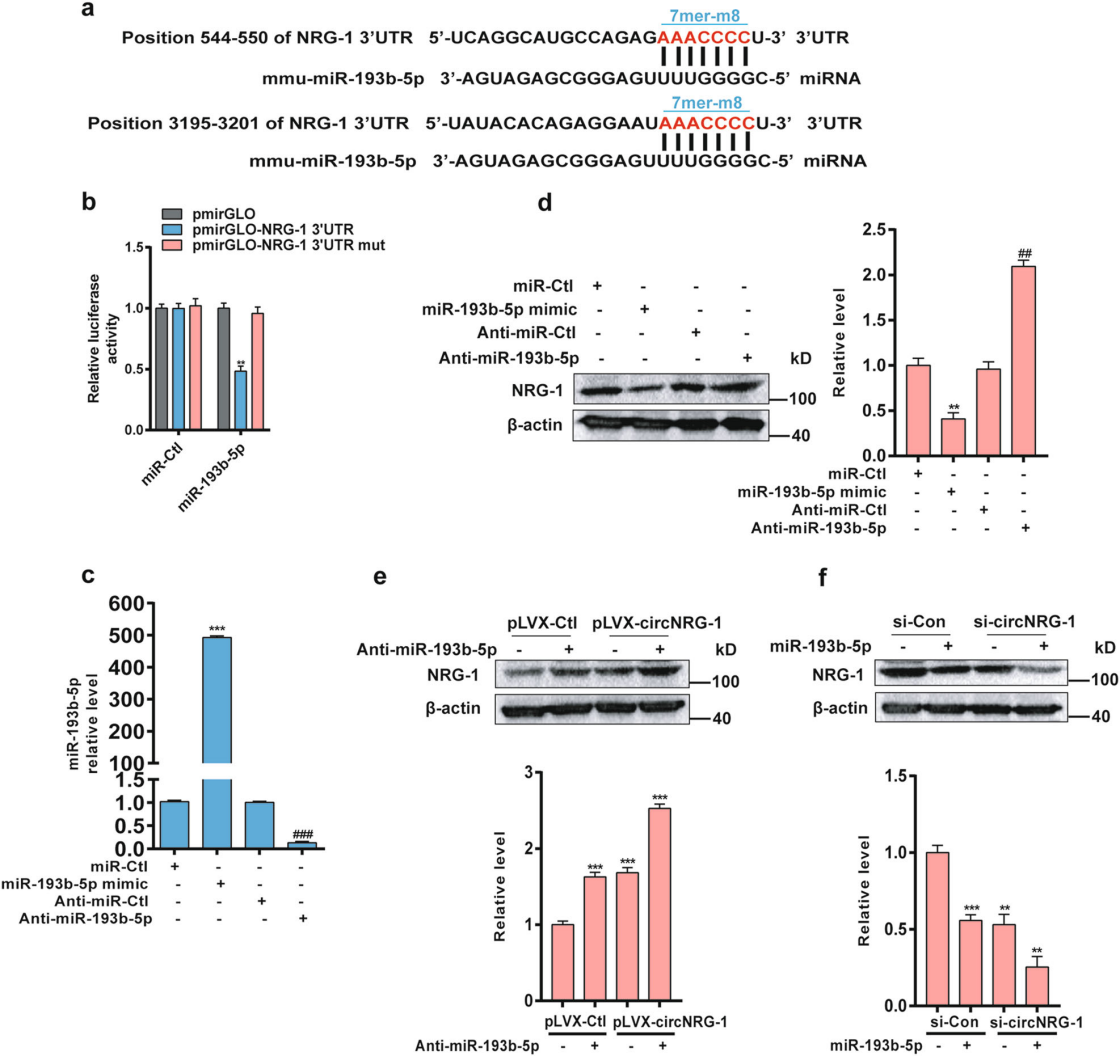
miR-193b-5p inhibits NRG-1 expression by absorbing its 3'-UTR in MAMSCs. **a** The miR-193b-5p-binding sites in the 3'-UTR of NRG-1 mRNA are shown in red. **b** HEK 293A cells were co-transfected with miR-193b-5p mimic and the wild-type pmirGLO-NRG-1 3′-UTR or its mutant pmirGLO-NRG-1 3′-UTR mut. After 24 h, luciferase activities were measured. Data are the means ± SEM of three independent experiments. **P < 0.01 *vs*. pmirGLO or pmirGLO-NRG-1 3′-UTR mut. **c** qRT-PCR detection of miR-193b-5p expression in MASMCs transfected with miR-193b-5p mimic or anti-miR-193b-5p and their corresponding controls. Data represent the means ± SEM of three independent experiments. ***P < 0.001 *vs*. miR-Ctl, ###*P* < 0.001 *vs*. anti-miR-Ctl. **d** MASMCs were transfected with miR-193b-5p mimic or anti-miR-193b-5p, as well as with their corresponding controls. NRG-1 expression was analyzed by Western blot. The right panel shows densitometric analyses from three independent experiments. **P < 0.01 *vs*. miR-Ctl, ##*P* < 0.01 *vs*. Anti-miR-Ctl. **e** Western blot detected NRG-1 expression in MASMCs transfected with pLVX-circNRG-1, anti-miR-193b-5p, or both of them. NRG-1 expression was quantitated as described above (Down panel). ***P < 0.001 *vs*. pLVX-Ctl+Anti-miR-193b-5p-untreated group. **f** MASMCs were transfected with si-circNRG-1, miR-193b-5p, or both of them for 24 h. NRG-1 expression was analyzed by Western blot and was quantitated as described above (Down panel). **P < 0.01, ***P < 0.001 *vs*. si-Con+miR-193b-5p-untreated group

NRG-1 was regarded as a miRNA target based on a network in which circRNA-miRNA-mRNA was constructed using cytoscape. The predicted ceRNA mechanism was finally described: circNRG-1 acted as a sponge or ceRNA for miR-193b-5p, while NRG-1 was the target gene (Fig. 4). To see whether NRG-1 was a direct target of miR-193b-5p, we used a bioinformatics approach to search the potential matching site of miR-193b-5p in the NRG-1 3′-UTR, and found that mouse NRG-1 3′-UTR contained two miR-193b-5p-binding sites at nucleotides 544–550 and 3195–3201 (Fig. 5a). To evaluate the effect of miR-193b-5p on NRG-1 expression, we constructed the wild-type pmirGLO-NRG-1 3'-UTR and its mutant pmirGLO-NRG-1 3′-UTR mut and co-transfected HEK 293A cells with them and miR-193b-5p mimic or miR-Ctl. The luciferase assay showed that miR-193b-5p mimic, but not control oligonucleotide (miR-Ctl), decreased the luciferase activity by 50%, whereas the mutation of the miR-193b-5p-binding site in the NRG-1 3′-UTR completely restored luciferase activity in the presence of the miR-193b-5p mimic (Fig. 5b). In the subsequent experiment, we transfected MASMCs with miR-193b-5p mimic and inhibitor anti-miR-193b-5p (Fig. 5c) and confirmed that the mimic and its antagomir markedly reduced or increased the NRG-1 protein expression, respectively, as compared with their corresponding controls (Fig. 5d). In addition, circNRG-1 overexpression and miR-193b-5p knockdown alone, or in combination in particular, significantly increased the NRG-1 expression level (Fig. 5e). In contrast, knockdown of circNRG-1 plus miR-193b-5p mimic worked together to decrease the NRG-1 expression level (Fig. 5f). These findings indicated that miR-193b-5p inhibited the NRG-1 expression in MASMCs by targeting its 3′-UTR, and that circNRG-1 facilitated the NRG-1 expression by absorbing miR-193b-5p.

### Ang II inhibits apoptosis via down-regulating NRG-1 expression in MASMCs

**Fig. 6:**
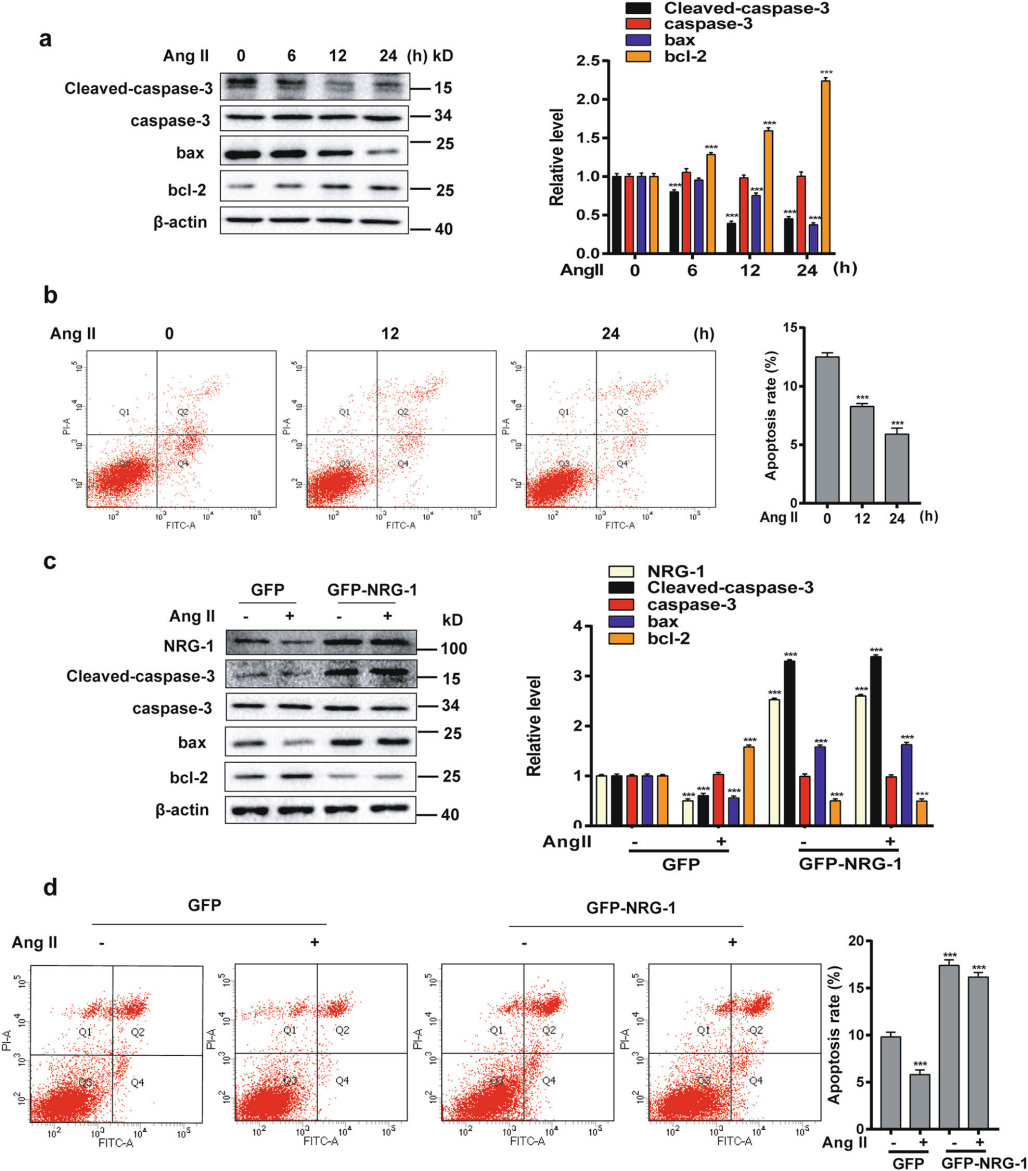
NRG-1 participates in the inhibition of apoptosis of MASMCs by Ang II. **a** MASMCs were treated with Ang II (10^−7^ M) for indicated times. Cleaved-caspase-3, caspase-3, bax and bcl-2 expression levels were detected by Western blot. The right panel shows densitometric analysis from three independent experiments. ***P < 0.001 *vs*. 0 h. **b** MASMCs were treated with different times of Ang II (10^−7^ M) and then the apoptotic rate was determined by flow cytometry analysis using annexin V/propidium iodide double staining. The right panel shows the apoptotic ratio from three independent experiments. ***P < 0.001 *vs*. 0 h. **c** MASMCs were infected with Ad-GFP-NRG-1 or Ad-GFP for 24 h and then treated with or without Ang II (10^−7^ M) for an additional 12 h, Western blot detected NRG-1, Cleaved-caspase-3, caspase-3, bax and bcl-2 levels. Data represent the means ± SEM of three independent experiments. ***P < 0.001 *vs*. Ad-GFP+Ang II-untreated group. **d** The apoptotic rate was determined and quantitative analysis for the flow cytometry experiment is presented on the right panel. Data represent the means ± SEM, *n* = 3. ***P < 0.001 *vs*. Ad-GFP+Ang II-untreated group

To examine the role of Ang II in MASMC apoptosis, we used Western blot to examine proteins related to apoptosis and anti-apoptosis. The apoptosis proteins Cleaved-caspase-3 and bax were decreased and the anti-apoptosis protein bcl-2 was increased in a time-dependent manner with Ang II (10^−7^ M), but the caspase-3 protein level remained unchanged significantly (Fig. 6a). To further confirm the ability of Ang II to confer resistance to apoptosis, flow cytometry combined with annexin V/propidium iodide (PI) staining was used to assess apoptosis. As suggested in Fig. 6b, the apoptotic rate of MASMCs induced by Ang II (10^−7^ M) decreased from 12% to 6%. These results indicated that Ang II attenuated apoptosis in MASMCs. To verify whether NRG-1 participated in Ang II-reduced apoptosis in MASMCs, we overexpressed NRG-1 through transfecting adenovirus vectors with or without Ang II. The results showed that overexpression of NRG-1 could reverse the expression levels of Cleaved-caspase-3, bax and bcl-2 (Fig. 6c). In addition, the apoptotic rate of overexpression NRG-1 was reversed from 9.7% to 17.4% (Fig. 6d). In conclusion, up-regulation of NRG-1 abrogated the effect of Ang II on the inhibition of MASMC apoptosis.

## Discussion

This study revealed that (1) Ang II decreased NRG-1 expression, including NRG-1-ECD and NRG-1-ICD in MASMCs; (2) Ang II down-regulated circNRG-1 expression, and circNRG-1 acted as a sponge binding miR-193b-5p, which targeted the 3′-UTR of NRG-1 mRNA, to regulate NRG-1 expression; and (3) Ang II inhibited apoptosis of MASMCs by reducing the NRG-1 expression. Based on the above findings, we concluded that the circNRG-1/miR-193b-5p/NRG-1 axis regulated the anti-apoptotic effect of MASMCs caused by Ang II.

It has been shown that endocardial endothelia and small subendocardial vessels are the primary cell source of NRG-1, and NRG-1 and its receptor ErbB play crucial roles in cardiac development, structural maintenance, and functional integrity of the heart^28^. Although our previous study found that TGF-β1 and PDGF-BB increased and decreased NRG-1 expression in HASMCs^15^, respectively, whether and how Ang II regulated NRG-1 in MASMCs remained unknown. The present data support the idea that Ang II decreases the NRG-1 protein expression in MASMCs and down-regulates it post-transcriptionally. NRG-1/ErbB signaling is an important potential mechanism for regulating vascular remodeling^29^. Hedhli et al. found that ErbB_2_, ErbB_3_ and ErbB_4_ were expressed in HASMCs^30^. Interestingly, we found that full-length NRG-1, NRG-1-ECD and NRG-1-ICD were all down-regulated by Ang II, p-ErbB_2_ and p-ErbB_4_ expression levels were also decreased in a time-dependent manner after stimulation by Ang II (Supplementary Fig. S1). This means that reduced phosphorylation of ErbB receptors was due to the low expression of NRG-1-ECD.

Notably, we found that Ang II down-regulated NRG-1 protein expression without affecting the mRNA level in MASMCs, which urged us to make clear whether some miRNAs or circRNAs regulated the NRG-1 expression at the post-transcriptional level. According to the circRNA microarray, we found that circNRG-1 was also down-regulated by Ang II, and this circRNA was not on the list of circBase, meaning that circNRG-1 is a new circRNA, so we used RT-PCR and Sanger sequencing to confirm whether circNRG-1 was derived from NRG-1 gene. In addition, we found that circNRG-1 overexpression or knockdown could increase or decrease the NRG-1 expression, so we wondered whether this mechanism of circNRG-1 was involved in the regulation of NRG-1 protein level. To simplify this problem, we tried to use the ceRNA theory^31^ to explain how circNRG-1 exerted its effect through bioinformatic analysis by establishing a circRNA-miRNA-mRNA regulatory model based on the ceRNA theory. As expected, we found that circNRG-1 acted as a miR-193b-5p sponge and interacted with miR-193b-5p to target the 3'-UTR of NRG-1 mRNA, thus increasing the NRG-1 expression in MASMCs.

In general, Ang II has been shown to induce or prevent apoptosis in several cell types^32,33^ but the role of Ang II in regulating apoptosis of VSMCs remains controversial. Here, we firstly demonstrated a novel mechanism of Ang II in inducing MASMCs to exhibit apoptosis resistance. The results showed that Ang II (10^−7^ M) decreased the apoptosis gene Cleaved-caspase-3 and bax, and increased the anti-apoptosis gene bcl-2 in MASMCs. In addition, the apoptotic rate of MASMCs was reduced by about 50%. This is similar to the finding in a previous study that Ang II inhibited VSMC apoptosis^8^, but different from the result of other studies that Ang II promoted apoptosis of VSMCs^34^. This may be due to different concentrations of Ang II used, or the different receptors activated by Ang II. In the early stages of vascular remodeling, a low Ang II environment may inhibit VSMC apoptosis, but with the phase advancing, the high Ang II environment stimulated VSMC apoptosis. In addition, Ang II exerted its biological effect through AT_1_R and AT_2_R, which are known to be associated with proliferation and apoptosis^35,36^. Several studies demonstrated that AT_1_R activation resulted in proliferation and anti-apoptosis whereas AT_2_R activation induced apoptosis in VSMCs^37^. In the present study, we blocked AT_1_R or AT_2_R to observe the apoptotic rate of MASMCs, and found that AT_1_R blockage could reverse the apoptotic rate of Ang II, but not the AT_2_R inhibitor (Supplementary Fig. S2). Finally, Ang II-induced anti-apoptosis was shown to be dose-dependent, and AT_1_R signaling was found to be a decisive response.

To further investigate the role of NRG-1 in Ang II-induced apoptosis resistance, we overexpressed NRG-1 and found that higher expression of NRG-1 reversed the inhibitory effect of Ang II on apoptosis in MASMCs. Together, these data suggest NRG-1 as a critical factor for mediating the resistance to apoptosis conferred by Ang II in MASMCs, and NRG-1 may be a potential therapeutic target for vascular remodeling associated with RAAS overactivation in atherosclerosis, hypertension and vascular diseases.

## Supplementary information

Supplementary Figure S1.

Supplementary Figure S2.

Supplementary Figure legends.
