# Supplementary figures and images for: Angiotensin II inhibits apoptosis of mouse aortic smooth muscle cells through regulating the circNRG-1/miR-193b-5p/NRG-1 axis
Source: Cell Death Dis. 2019 May 1;10(5):362. doi: 10.1038/s41419-019-1590-5 (PMC6494886; doi:10.1038/s41419-019-1590-5)

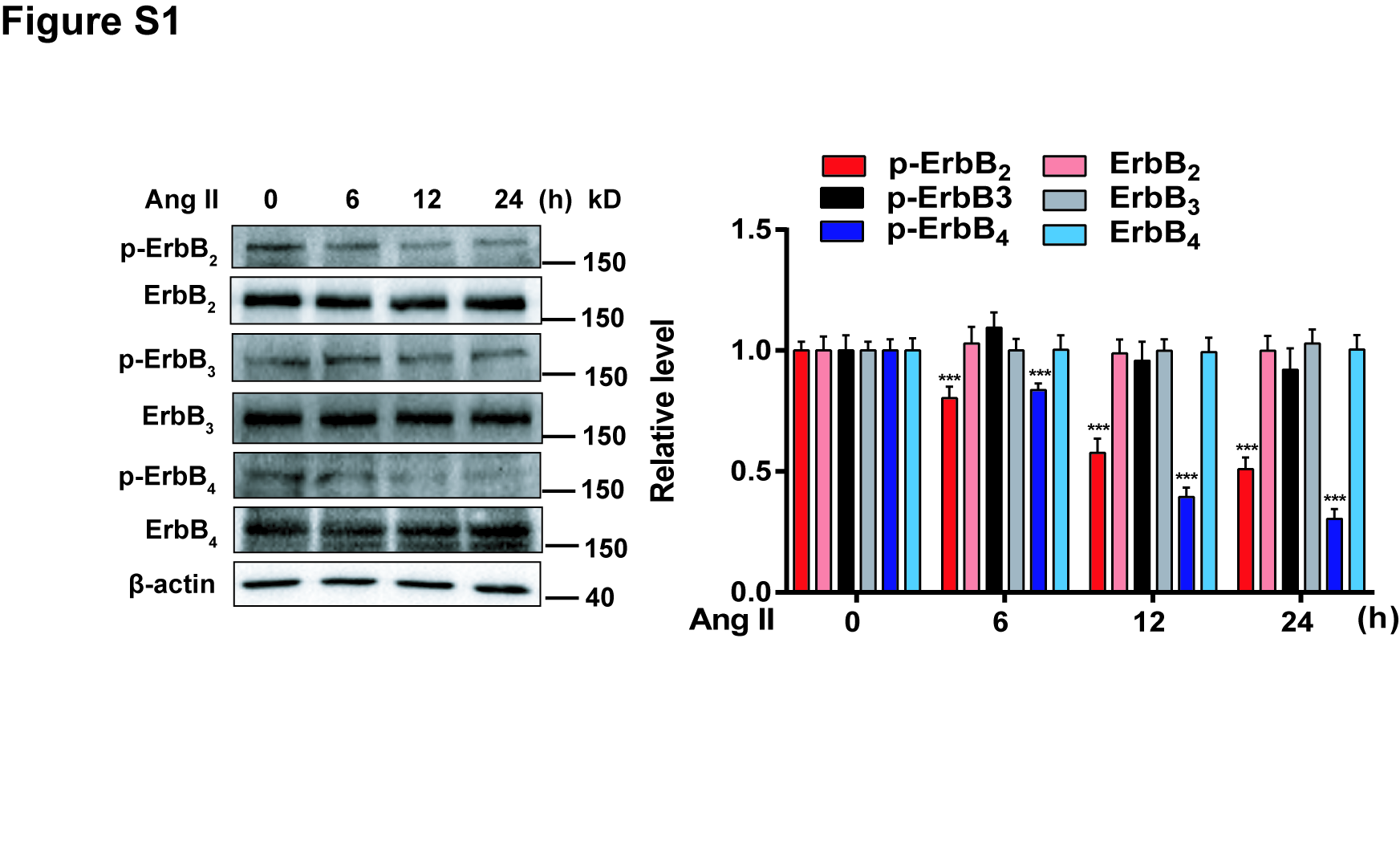

Supplement: Supplementary file 1 — Supplementary Figure S1. [file 41419_2019_1590_MOESM1_ESM.tif]

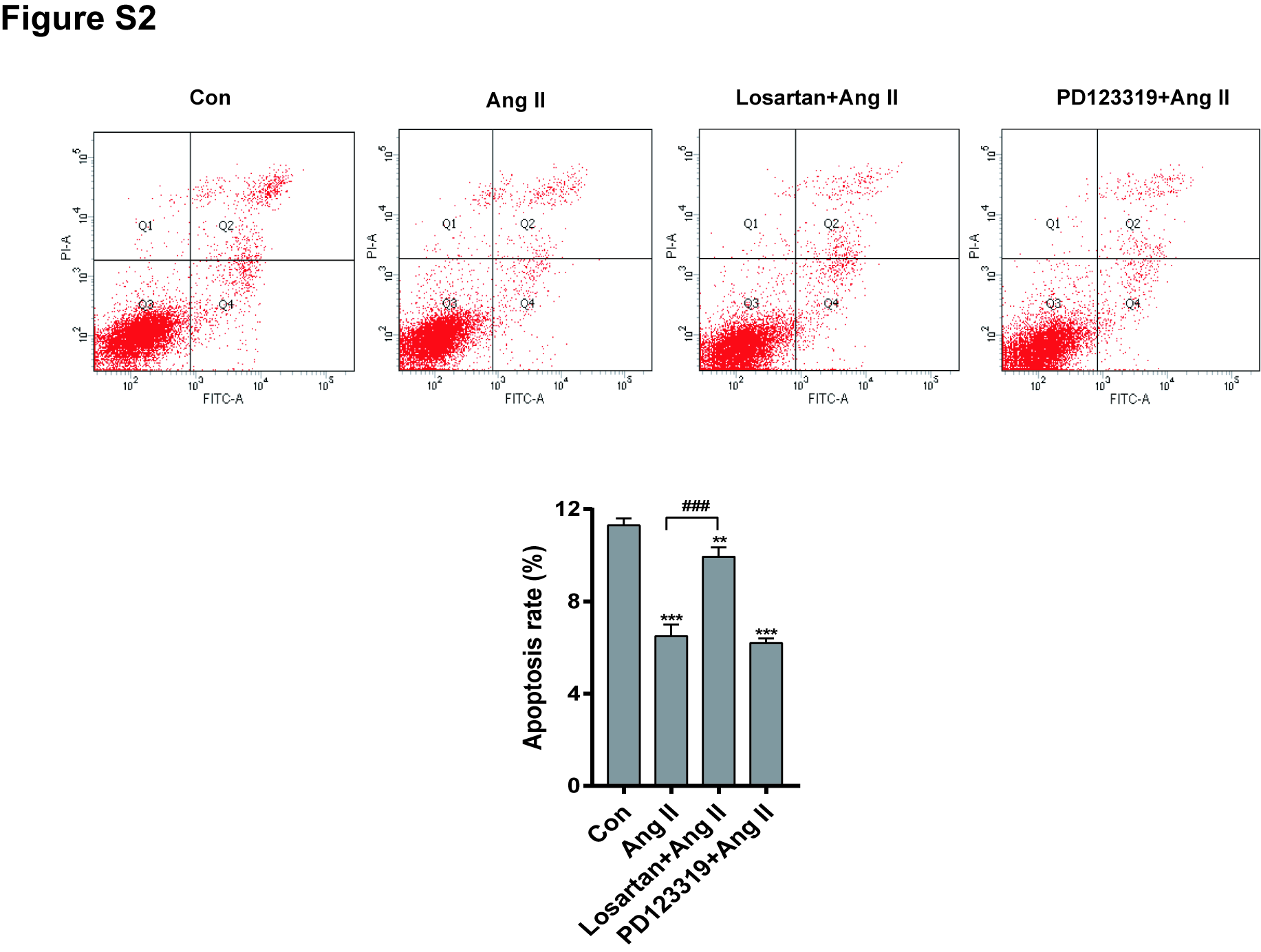

Supplement: Supplementary file 2 — Supplementary Figure S2. [file 41419_2019_1590_MOESM2_ESM.tif]
