# Supplementary material for: Angiotensin II inhibits apoptosis of mouse aortic smooth muscle cells through regulating the circNRG-1/miR-193b-5p/NRG-1 axis
Source: Cell Death Dis. 2019 May 1;10(5):362. doi: 10.1038/s41419-019-1590-5 (PMC6494886; doi:10.1038/s41419-019-1590-5)
Supplement: Supplementary file 3 — Supplementary Figure legends. [file 41419_2019_1590_MOESM3_ESM.docx]

**Legends**

**Figure. S1 Ang II decreases the p-ErbB_2_ and p-ErbB_4_ expression levels in MASMCs.** MASMCs were treated with Ang II (10^-7^ M) for indicated times. p-ErbB_2_, ErbB_2,_ p-ErbB_3_, ErbB_3,_ p-ErbB_4_ and ErbB_4_ expression levels were detected by Western blot. The right panel shows densitometric analysis from three independent experiments. ***P<0.001 *vs.* 0 h.

**Figure. S2 AT_1_R inhibitor blocks the anti-apoptotic effect of Ang II in MASMCs.** MASMCs were treated with different stimuli and then cell apoptotic rate was determined by flow cytometry analysis using annexin V/propidium iodide double staining. The down panel shows the apoptotic rate from three independent experiments. **P<0.01, ***P<0.001 *vs.* Con, ###P<0.001 *vs.* Ang II.
